# Altered Bone Development and an Increase in FGF-23 Expression in *Enpp1^−/−^* Mice

**DOI:** 10.1371/journal.pone.0032177

**Published:** 2012-02-16

**Authors:** Neil Charles Wallace Mackenzie, Dongxing Zhu, Elspeth M. Milne, Rob van 't Hof, Aline Martin, Darryl Leigh Quarles, José Luis Millán, Colin Farquharson, Vicky Elisabeth MacRae

**Affiliations:** 1 The Roslin Institute and Royal (Dick) School of Veterinary Studies, The University of Edinburgh, Midlothian, Scotland, United Kingdom; 2 Rheumatic Diseases Unit, Molecular Medicine Centre, Institute of Genetics and Molecular Medicine, University of Edinburgh, Edinburgh, United Kingdom; 3 Sanford Children's Health Research Center, Sanford-Burnham Medical Research Institute, La Jolla, California, United States of America; 4 University of Tennessee Health Science Center, Memphis, Tennessee, United States of America; University of Munich, Germany

## Abstract

Nucleotide pyrophosphatase phosphodiesterase 1 (NPP1) is required for the conversion of extracellular ATP into inorganic pyrophosphate (PP_i_), a recognised inhibitor of hydroxyapatite (HA) crystal formation. A detailed phenotypic assessment of a mouse model lacking NPP1 (*Enpp1^−/−^*) was completed to determine the role of NPP1 in skeletal and soft tissue mineralization in juvenile and adult mice. Histopathological assessment of *Enpp1^−/−^* mice at 22 weeks of age revealed calcification in the aorta and kidney and ectopic cartilage formation in the joints and spine. Radiographic assessment of the hind-limb showed hyper-mineralization in the talocrural joint and hypo-mineralization in the femur and tibia. MicroCT analysis of the tibia and femur disclosed altered trabecular architecture and bone geometry at 6 and 22 weeks of age in *Enpp1^−/−^* mice. Trabecular number, trabecular bone volume, structure model index, trabecular and cortical thickness were all significantly reduced in tibiae and femurs from *Enpp1^−/−^* mice (P<0.05). Bone stiffness as determined by 3-point bending was significantly reduced in *Enpp1^−/−^* tibiae and femurs from 22-week-old mice (P<0.05). Circulating phosphate and calcium levels were reduced (P<0.05) in the *Enpp1^−/−^* null mice. Plasma levels of osteocalcin were significantly decreased at 6 weeks of age (P<0.05) in *Enpp1^−/−^* mice, with no differences noted at 22 weeks of age. Plasma levels of CTx (Ratlaps™) and the phosphaturic hormone FGF-23 were significantly increased in the *Enpp1^−/−^* mice at 22 weeks of age (P<0.05). *Fgf-23* messenger RNA expression in cavarial osteoblasts was increased 12-fold in *Enpp1^−/−^* mice compared to controls. These results indicate that *Enpp1^−/−^* mice are characterized by severe disruption to the architecture and mineralization of long-bones, dysregulation of calcium/phosphate homeostasis and changes in *Fgf-23* expression. We conclude that NPP1 is essential for normal bone development and control of physiological bone mineralization.

## Introduction

Bone development and remodelling throughout life occurs through a tightly controlled balance of osteoblastic bone formation and resorption by osteoclasts. Bone formation during development and the remodeling cycle are a result of the secretion of proteins of the bone extracellular matrix (ECM), or osteoid and its mineralization in a two-stage process. Primary mineralization is a rapid phase where 70% of complete mineralization occurs. In contrast, secondary mineralization occurs more slowly and is characterized by a gradual maturation of the mineral and is essential for the hardness and rigidity that enables the skeleton to resist gravitational and mechanical loading. During the resorption phase of the remodeling cycle, osteoclasts through acid production and protease secretion induce demineralization and degradation of the bone matrix [1], [2].

Mineralization is initiated within osteoblast- and chondrocyte-derived matrix vesicle (MVs) where Ca^2+^ ions and inorganic phosphate (P_i_) crystallize to form hydroxyapatite (HA) [3]. The MVs then release HA into the ECM, where further crystal growth occurs [4], [5]. The mineralization process depends on a regulated balance of various physiochemical and protein inducers and inhibitors. Physiochemical factors include calcium concentrations and pH, as well as the regulation of ECM mineralization inhibitors such as inorganic pyrophosphate (PP_i_), and inducers such as in inorganic phosphate (P_i_). The ratio of P_i_ to PP_i_ controls the deposition of bone mineral and concentrations of these factors are regulated by tissue-non-specific alkaline phosphatase (TNAP), ecto-nucleotide pyrophosphatase/phosphodiesterase-1 (NPP1) and the ankylosis protein (ANK) [6]–[10]. In addition ECM proteins, such as dentin matrix protein 1 (DMP1) [11], matrix gla protein, osteopontin (OPN) [12]–[14] and phosphate regulating endopeptidase homolog, X-linked (PHEX) [15], play important roles in regulating the mineralization process. Furthermore, primary alterations in bone mineralization in hereditary hypophosphatemic disorders caused by mutations of *Phex* and *Dmp1* as well as mutations of *Enpp1* [16], [17] and *Ank* [18] have been associated with increased circulating levels of the bone-derived phosphaturic factor fibroblast growth factor 23 (FGF-23), suggesting that bone metabolism is linked to systemic phosphate homeostasis.

NPP1 (EC 3.1.4.1) is a plasma membrane glycoprotein that ectoplasmically generates PP_i_, a recognised inhibitor of HA crystal formation [19], from nucleoside triphosphates [20]. Intracellular to extracellular channelling of PP_i_ is mediated by ANK [21], [22]. Extracellular PP_i_ concentration is regulated by TNAP, which hydrolyzes PP_i_ in the ECM to release P_i_ and establishing a P_i_/PP_i_ ratio permissive for the formation of HA crystals [23]–[26]. Further feedback signalling allows mediation of the mineralization process; both P_i_ and PP_i_ inhibits the enzymatic activity of TNAP [19], and both exogenous P_i_ and PP_i_ induce osteopontin (OPN) [7], [9], [19]. It has been widely reported that lack of NPP1 function is associated with a reduction in levels of circulating PP_i_ [7], [27], [28].

Vascular calcification is a highly regulated cellular process similar to skeletal mineralization [29], [30]. By maintaining high levels of extracellular PP_i_ soft tissues, particularly vascular cells and articular cartilage, can suppress spontaneous calcification [31]. In human infants, severe NPP1 deficiency is associated with a syndrome of spontaneous infantile arterial and periarticular calcification [32], [33]. Elevated levels of FGF-23, an inhibitor of renal P_i_ re-absorption, have been observed in patients suffering from hypophosphatemic rickets as a result of a loss of function mutation in the NPP1 gene. This indicates that NPP1 may also have a significant role in phosphate homeostasis [17].

In naturally occurring mouse models, the link between defective NPP1 expression and altered mineralization was initially demonstrated in “tiptoe walking” (*ttw/ttw*) mice. These animals are homozygous for a G→T substitution resulting in the introduction of a stop codon in the NPP1 coding sequence. The subsequent truncated protein leads to the loss of a vital calcium binding domain and two putative glycosylation sites [34]. The *ttw/ttw* mouse phenotype includes the postnatal development of progressive ankylosing intervertebral and peripheral joint hyperostosis, as well as spontaneous arterial and articular cartilage calcification and increased vertebral cortical bone formation [34]–[38]. Transgenic mice that are homozygous for a disruption in Exon 9 of the *Enpp1* gene exhibit abnormalities that are almost identical to those present in naturally occurring *ttw/ttw* mice [27]. These include decreased levels of extracellular PP_i_, with phenotypic features including significant alterations in bone mineralization in long bones and calvariae, and pathologic, severe peri-spinal soft tissue and arterial calcification [7], [9], [28].

To date the examination of the role of NPP1 in bone function has been limited to the study of immature 10-day-old mice [28]. However, little is known about its role in the maintenance of the skeleton during the aging process. Therefore, we have studied juvenile and adult mice to determine the effects of NPP1 on skeletal maturation, which may not be apparent in the immature developing skeleton. These studies have confirmed that the structural and mechanical properties of the *Enpp1^−/−^* adult skeleton are more severely compromised than their juvenile counterparts. Furthermore, our analysis of *Enpp1^−/−^* mice discloses an increase in circulating FGF-23 and *Fgf-23* mRNA expression in the calvaria indicating a possible role for *Enpp1* in phosphate regulation, a finding consistent with recent human genetic studies [16], [17], [39].

## Materials and Methods

### Maintenance of Enpp1^−/−^ mice

The generation and characterization of *Enpp1* ^−/−^ mice has been previously described [33]. Genotyping was done on genomic DNA isolated from ear clips and analyzed using PCR protocols developed by Genetyper (Genetyper, New York, USA). Male and female mice were culled at 6 weeks (juvenile) and 22 weeks (adult) of age; gender and number of mice studied are specified in each individual experiment. All animal experiments were approved by The Roslin Institute's Animal Users Committee and the animals were maintained in accordance with UK Home Office guidelines for the care and use of laboratory animals.

### Gross analysis

Following euthanasia of male and female knockout (*Enpp1^−/−^)* and wild-type mice at 6 and 22 weeks of age, body length (crown – rump) and body weight measurements were recorded (n = 8). Radiographic assessment of the left hind-limb was made from X-ray images (Faxitron, Wheeling, IL, USA). Thereafter, femur and tibia length and width were measured using DigiMax digital vernier callipers (R. S. Components Ltd, Corby, Northants, UK).

### Preparation of tissue for microscopical analysis

Heart, aorta and kidney were fixed in 10% neutral buffered formalin (NBF), embedded in paraffin wax and 4 µM thick sections were stained with H&E, alizarin red and von Kossa to assess calcification status. Similarly, long bones and femorotibial and talocrural joints were fixed in 10% NBF and either decalcified in 10% EDTA for 14 days at 4°C and embedded in ax or directly embedded in methyl-methacrylate (MMA) according to standard procedures. Longitudinal plastic sections were cut at 5 µm using a Leica microtome.

### RNA isolation and RT-qPCR analysis of Fgf-23 expression

Total RNA were extracted from homogenized calvarial bone from 12-week-old mice using TRI Reagent (Molecular Research Center, Cincinnati, OH) and then treated with RNase-free DNase (Qiagen, Valencia, CA). First-strand cDNA was synthesized using iScript cDNA Synthesis Kit (Bio-Rad, Hercules, CA). Total RNA (1 µg) was used in each 20 µl reverse transcriptase reaction. The iCycler iQ Real-Time PCR Detection System and iQ SYBR Green Supermix (Bio-Rad) were used for real-time quantitative PCR analysis. The expression was normalized to glyceraldehyde-3-phosphate dehydrogenase (GAPDH) in the same sample and expressed as 100% of the control (wild-type). Sequences of primers used for real-time quantitative RT-PCR of *Fgf-23* were FGF23.foward CAC TGC TAG AGC CTA TTC and FGF23.reverse CAC TGT AGA TAG TCT GAT GG, GAPDH Forward AAT GGG GTG AGG CCG GTG CT and GAPDH Reverse GCA GTG ATG GCA TGG ACT GTG GT.

### Histomorphometric analysis

The width of the proximal growth plate of both tibiae and femurs was determined using image analysis software (Nikon, Kingston upon Thames, Surrey, UK) on toluidine blue stained paraffin sections. Growth plate width was determined at 10 different points along the breadth of the growth plate in two sections from each bone [40]. MMA embedded sections of the tibiae taken from 22-week-old female wild-type (n = 4) and *Enpp1* ^−/−^ mice (n = 4) were reacted for tartrate resistant acid phosphatase (TRAcP) activity to visualize osteoclasts and counterstained with aniline blue to visualize bone. For the analysis of osteoblasts, sections were reacted for alkaline phosphatase activity using Naphthol AS-MX Phosphate as substrate and Fast Violet B salt (Sigma-Aldrich, Gillingham, UK). Sections were viewed using a 4× or 10× objective lens on a Zeiss Axioimager (Zeiss, Jena, Germany) microscope and images of the proximal tibia were captured using a QImaging Retiga 4000R camera. Osteoclast number and resorption surfaces were determined using in-house software based on ImageJ (developed by Rob van 't Hof).

### Micro-computed tomography

Tibiae and femors were dissected from 6 and 22-week-old male and female wild-type and *Enpp1^−/−^* mice and stored in distilled water at −20°C (n = 8). The bones were scanned using a micro-computed tomography (µCT) system (Skyscan 1172 X-Ray microtomograph, Aartselaar, Belgium) to evaluate trabecular architecture and cortical bone geometry. High-resolution scans with an isotropic voxel size of 5 µm were acquired (60 kV, 0.5 mm aluminium filter, 0.5° rotation angle). Two images were averaged at each rotation angle to reduce signal noise and improve the accuracy of the BMD measurements. Scan time was approximately 30 min per bone. The scans were reconstructed using NRecon software (Skyscan, Belgium). For each bone, a 1000 µm section of the metaphysis was taken for analysis of trabecular bone, using the base of the growth plate as a standard reference point. A further 1500 µm below the base of the metaphysis section a 400 µm section of the mid-diaphysis was scanned for analysis of cortical structure.

Noise in the reconstructed images was reduced by applying a median filter (radius = 1), and bone tissue was identified by thresholding. The optimal threshold was determined from the image histograms, and was set to exclude soft tissue, but to include poorly mineralised bone. The same threshold was used in all samples. The thresholded image was used as a mask to measure the BMD of the bone structures, using the unfiltered image data as input. For accurate calculation of BMD appropriate calibration of the Skyscan CT analyser was carried out with known density calcium hydroxyapatite phantoms.

The following parameters were analyzed using CTAn software (Skyscan, Belgium); in trabecular bone, percent bone volume (% BV/TV), trabecular number (Tb.N; /mm), bone mineral density (BMD; g/cm^3^), trabecular thickness (Tb.Th; mm), trabecular separation (Tb.Sp) and structure model index (SMI) were evaluated. In cortical bone, % BV/TV, BMD (g/cm^3^), cortical thickness, cross-sectional area (mm^2^), percentage of closed pores and polar moment of inertia (mm^4^) were evaluated.

### Mechanical testing

3-point bending for the determination of bone stiffness and breaking strength was completed using a Lloyd LRX5 materials testing machine (Lloyd Instruments, West Sussex, UK) fitted with a 2 kN load cell [41]. Tibiae and femora from 6 and 22-week-old male and female wild-type and *Enpp1* ^−/−^ mice (n = 7) were tested to fracture. The span was fixed at 5.12 mm for femora and at 6.95 mm for tibiae. The cross-head was lowered at 1 mm/min and data were recorded after every 0.2 N change in load and every 0.1 mm change in deflection. Failure and fracture points were identified from the load-extension curve as the point of maximum load and where the load rapidly decreased to zero, respectively. The maximum stiffness was defined as the maximum gradient of the rising portion of this curve, and the yield point was the point at which the gradient reduced to 90% of this value. Both values were calculated from a polynomial curve fitted to the rising region of the load-extension curve in Sigmaplot (Systat Software Inc., San Jose, USA).

### Whole blood and plasma analysis

Immediately following euthanasia, blood samples from 22-week-old wild-type and *Enpp1* ^−/−^ female mice (n = 10) were obtained by cardiac puncture and collected into lithium heparin tubes from which whole blood and plasma samples were prepared. Haematological analysis of whole blood samples was undertaken using a Pentra 60 impedance haematology analyser (Horiba Medical, Northampton, UK) for determination of red blood cell count, packed cell volume, haemoglobin concentration, mean corpuscular volume (MCV), mean corpuscular haemoglobin concentration (MCHC) and white blood cell (WBC) count. Differential WBC counts were carried out manually on Wright's-stained smears and the percentage and absolute number of neutrophils, lymphocytes, monocytes, eosinophils and basophils were recorded.

To determine difference in bone remodelling rates, bone formation and resorption markers were measured in plasma samples taken from male mice at 6 and 22 weeks of age (n = 6). This was done using a sandwich ELISA osteocalcin kit (Mouse Osteocalcin EIA Kit; Biomedical Technologies Inc, Stoughton, MA, USA) and a C-terminal telopoptides of type I collagen ELISA kit (RatLaps TM, IDS, Boldon, UK) respectively, and performed according to manufacturer's instructions. Circulating FGF-23 levels were measured using an FGF-23 ELISA kit (Kainos Laboratories Inc., Tokyo, Japan). Inorganic phosphate, creatine kinase activity, TNAP activity, total protein, albumin, bile acids, cholesterol, creatinine, alanine aminotransferase and non-esterified fatty acids were measured on an IL600 biochemistry analyzer (Instrumentation Laboratory, Warrington, Cheshire, UK) using standard photometric kit methods (Instrumentation Laboratory). Total calcium, potassium and sodium were measured using the IL600 ISE (Instrumentation Laboratory). Globulin concentration was calculated by subtraction of the albumin from the total protein concentration.

### Statistical analysis

General linear model analysis, Student's t-test, Mann-Whitney non-parametric test and Pearson's correlations were used to assess the data where appropriate. All data are expressed as the mean +/− S.E.M. Statistical analysis was performed using Minitab 15 (Minitab Ltd, Coventry, UK), and confirmed using SPSS (IBM Software, New York, USA). *P*<0.05 was considered to be significant.

## Results

### Enpp1^−/−^ mice show reduced growth

Initial studies addressed whether *Enpp1* ^−/−^ mice displayed a reduced growth phenotype resembling that previously reported for 10-day-old *Enpp1^−/−^* mice. At 6 weeks of age a significant reduction in body weight was observed in both male (83.7%; P<0.05) and female (87.7%; P<0.01) *Enpp1* ^−/−^ mice (Table 1). A concomitant decrease in body length was also noted in male (87.3%; P<0.01) and female (89.2%; P<0.01) *Enpp1* ^−/−^ mice (Table 1). However, there were no significant differences in the lengths of the tibiae or femora of 6-week-old *Enpp1* ^−/−^ mice (Table 1). Normal long bone growth in juvenile *Enpp1* ^−/−^ mice was also confirmed by growth plate width analysis, which was similar in both *Enpp1* ^−/−^ and WT mice (data not shown). Detailed analysis of 22-week-old mice indicated that male and female *Enpp1* ^−/−^ mice were also significantly lighter (83.5%; P<0.001 and 65.7%; P<0.001 respectively) and shorter (91.7%; P<0.01 and 91.3%; P<0.01 respectively) (Table 1). The femurs of both male and female 22-week-old *Enpp1* ^−/−^ mice were significantly shorter than their WT counterparts (94%; P<0.05 and 96.1%; P<0.01). Interestingly the length of the tibia in the male 22-week-old *Enpp1* ^−/−^ mice was longer (103.1%; P<0.01), but there was no change seen in the 22-week-old females. No differences in femoral or tibial growth plate widths were observed at 22 weeks of age (data not shown).

**Table 1:** Body weight, body length and long bone length taken at 6 week and 22 weeks of age from male and female and *Enpp1^−/−^* and wild-type mice.

Sex	Age(weeks)	Genotype	Body weight (g)	Body length (cm)	Femur length (mm)	Tibia length (mm)
Female	6	*Enpp1^+/+^*	20.4 (0.5)	8.96 (0.12)	13.83 (0.01)	16.88 (0.07)
		*Enpp1^−/−^*	17.9 (0.5)**	7.99 (0.21)**	13.61 (0.17)	16.93 (0.09)
	22	*Enpp1^+/+^*	32.1 (0.7)	9.92 (0.13)	16.11 (0.42)	18.85 (0.25)
		*Enpp1^−/−^*	21.1 (0.7)***	9.06 (0.14)***	15.47 (0.13)**	19.26 (0.13)
Male	6	*Enpp1^+/+^*	25.2 (0.6)	9.79 (0.12)	14.52 (0.15)	17.57 (0.19)
		*Enpp1^−/−^*	21.1 (1.1)*	8.55 (0.27)**	14.00 (0.25)	17.18 (0.19)
	22	*Enpp1^+/+^*	31.0 (0.8)	9.72 (0.l8)	16.06 (0.16)	18.63 (0.11)
		*Enpp1^−/−^*	25.9 (1.0)***	8.91 (0.15)**	15.09 (0.59)*	19.21 (0.23)**

Data are presented as mean +/− SEM (n = 10). Significance is denoted by

*P<0.05,

**P<0.01.

***P<0.001.

### Histopathological features of Enpp1^−/−^ mice

**Figure 1:**
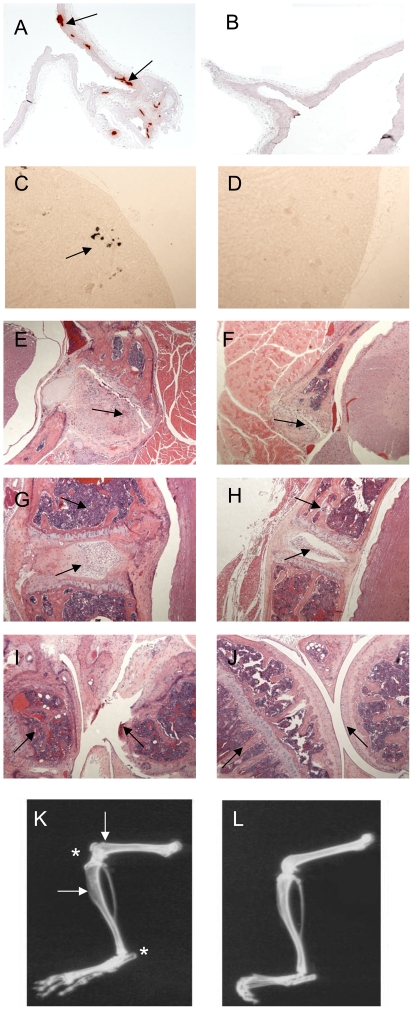
Histological and radiographic assessment of tissues from wild-type and *Enpp1^−/−^* mice. Calcification of the tunica media of the aorta was detected by alizarin red staining (arrows) in (A) *Enpp1^−/−^* compared to (B) wild-type control tissue. Ectopic calcification of the kidney was detected by von Kossa staining (arrow) in (C) *Enpp1^−/−^* compared to (D) wild-type control tissue. H&E stained transverse section of the neck of the (E) *Enpp1^−/−^* mouse showing an enlarged cervical vertebra and increased deposition of cartilage (arrows) compared to the (F) wild-type mouse. H&E stained longitudinal section of the mid-thoracic vertebrae of the *Enpp1^−/−^* mouse (G) showing a severely increased deposition of cartilage in the connective tissue and incursion into the spinal cord (arrows) compared to the (H) wild-type mouse. H&E stained sagittal section of the femorotibial joint of the (I) *Enpp1^−/−^* mouse showing remodelling of the femur and over-growth of ectopic cartilage (arrows) compared to the (J) wild-type mouse. Radiographs demonstrate calcification of the femorotibial and tarsocrural joint (asterisk), and reduced mineralization in the tibia and femur (arrows) of *Enpp1^−/−^* mice (K) compared to wild-type control (L).

In order to understand more fully the physiological role of NPP1 on skeletal development and pathological mineralization, a detailed histological assessment of adult *Enpp1* ^−/−^ mice at 22 weeks of age was undertaken. Alizarin red and von Kossa staining revealed abnormal soft tissue calcification in the coronary artery, the medial layer of the ascending aorta, aortic arch and brachiocephalic artery (Fig. 1A) and in the cortex of the kidney (Fig. 1C) of *Enpp1* ^−/−^ mice, compared to WT controls (Fig. 1B and 1D).

A striking hyperostosis (excessive bone growth) of the cervical (Fig. 1E) and thoracic (Fig. 1G) vertebrae, and the interphalangeal and femorotibial (Fig. 1I) joints was observed in *Enpp1* ^−/−^ mice compared to their wild-type counterparts (Fig. 1F, 1H and 1J respectively). Encroachment of bone lesions onto the spinal cord was observed, and may contribute to the abnormal gait observed in some *Enpp1* ^−/−^ mice. An increased presence of blood cells, thinner articular cartilage, misshapen and disorganised heads of the tibiae and femora and ectopic cartilage deposition were also noted in the femorotibial joint of the *Enpp1^−/−^* mice (Fig. 1I). Radiography of the hind-limb revealed increased mineralization of the knee and ankle joints, with reduced mineralization of the femur and tibia in the *Enpp1* ^−/−^ (Fig. 1K), compared to WT mice (Fig. 1L). These data show that lack of NPP1 activity significantly increases mineralization within the vertebrae and interphalangeal, femorotibial and talocrural joints, and indicate a reduction in long bone mineralization.

### Enpp1^−/−^ mice have reduced trabecular bone mass

*Enpp1^−/−^* mice have been reported to display reduced mineral content in both the growth plate and adjacent bone, with a reduction in bone volume fraction and trabecular thickness at 10 days of age [28]. We have now completed a comprehensive high-resolution µCT analysis of the tibia and femur to extend these observations by fully examining the effects of *Enpp1* deficiency on bone phenotype in male and female juvenile and adult mice.

**Figure 2:**
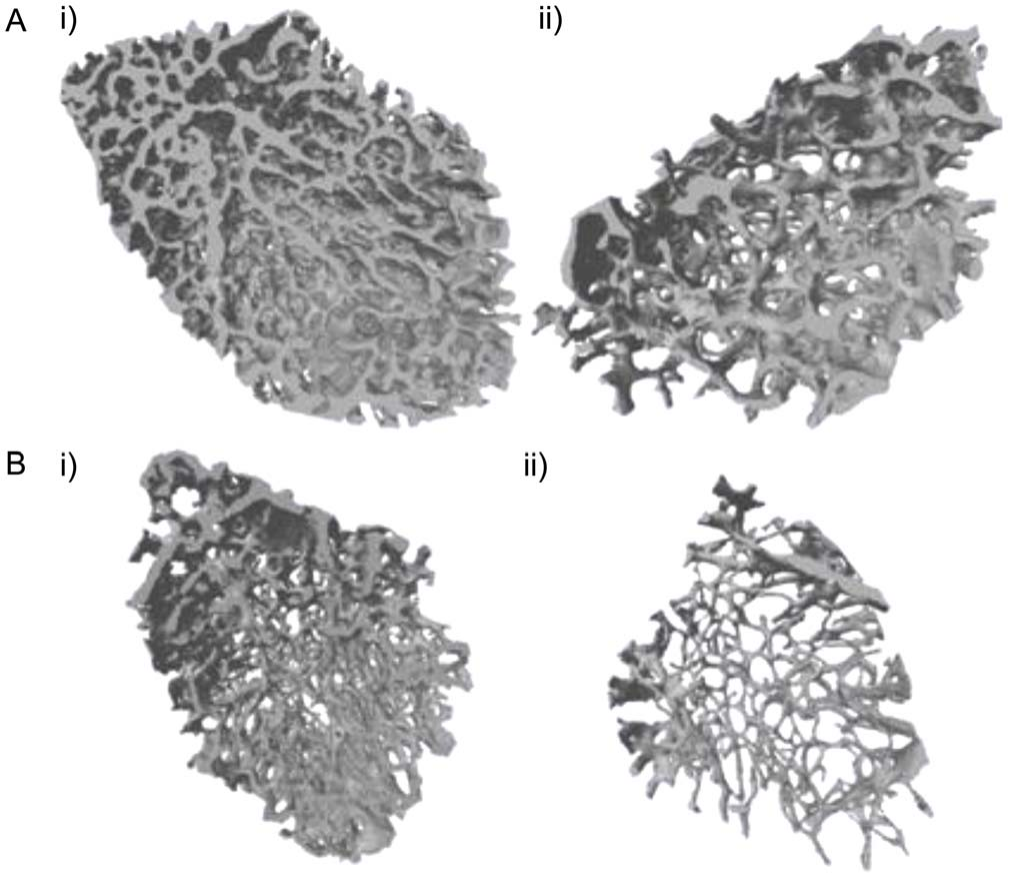
3D reconstruction of the trabecular bone scanned using microCT. (A) Wild-type and (B) *Enpp1^−/−^* mice femurs were collected at i) 6 weeks and ii) 22 weeks of age. These reconstructions illustrate the reduction of trabecular number in the *Enpp1^−/−^* mice.

Reduced bone volume in male *Enpp1^−/−^* mice was apparent in the trabecular compartment of the tibia and femur at both 6 and 22 weeks of age. *Enpp1^−/−^* mice had significantly reduced BV/TV, trabecular number and trabecular thickness compared to wild-type controls (Table 2; Fig. 2). These differences were more marked in the 22-week-old mice. Comparable changes were observed in female *Enpp1^−/−^* mice (data not shown). The structural model index (SMI), which quantifies the characteristic form of a 3D structure in terms of amounts of plates and rods [42], was also significantly higher in tibiae and femurs from male *Enpp1^−/−^* mice (P<0.05), with comparable changes in female mice (data not shown). This indicates that the trabeculae in *Enpp1^−/−^* mice are less ‘plate-like’ and less connected (Table 2). Trabecular BMD was unchanged in the *Enpp1^−/−^* mice. These results confirm that juvenile and adult *Enpp1^−/−^* mice have reduced trabecular bone and are consistent with previous reports of an association of an osteopenic phenotype in mice with *Enpp1* ablation [28].

**Table 2:** MicroCT analysis of trabecular bone in male wild-type and *Enpp1^−/−^* mice.

Bone	Age(weeks)	Genotype	% BV/TV	BMD (g/cm^3^)	Trab.Thickness (µm)	Trab. Number(TbN/µm)	Structural Model Index
Tibia	6	WT	19.75 (1.82)	1.04 (0.02)	40.81 (1.68)	0.0049 (0.0005)	1.60 (0.06)
		*Enpp1^−/−^*	12.53 (2.26)*	1.07 (0.03)	38.93 (1.97)	0.0032 (0.0005)*	1.85 ( 0.16)
	22	WT	14.46 (3.53)	1.17 (0.02)	53.84 (1.31)	0.0026 (0.0006)	1.93 (0.20)
		*Enpp1^−/−^*	4.78 (0.73)*	1.21 (0.02)	46.92 (1.08)***	0.001 (0.0002)*	2.52 (0.09)**
Femur	6	WT	28.26 (2.55)	1.04 (0.01)	45.91 (1.69)	0.0062 (0.0006)	0.98 (0.16)
		*Enpp1^−/−^*	16.08 (1.87)**	1.05 (0.01)	40.73 (1.31)*	0.0039 (0.0004)**	1.53 (0.19)*
	22	WT	15.09 (2.28)	1.12 (0.01)	53.34 (1.32)	0.0028 (0.0004)	1.85 (0.08)
		*Enpp1^−/−^*	4.75 (0.64)***	1.16 (0.02)	46.92 (1.52)**	0.001 (0.0001)***	2.54 (0.04)***

6 week femur (WT and *Enpp1^−/−^ n = 7*) and tibia (WT *n = 6*, *Enpp1^−/−^ n = 7*) and 22 week femur (WT and *Enpp1^−/−^ n = 9*) and tibia (WT and *Enpp1^−/−^ n = 9*) were tested. SEM is shown in brackets, significance is denoted by

*P<0.05,

**P<0.01.

***P<0.001.

### Enpp1^−/−^ mice have reduced cortical thickness

A significant reduction in cortical thickness was observed in the femur (P<0.05) and tibia (P<0.001) of male *Enpp1* null mice at 22 weeks of age (Table 3). Cortical thickness measured in 6-week-old mice was reduced but only significant in the femur (P<0.05). The cross-sectional area of cortical bone was also reduced in all samples, reaching significance in 22-week-old femurs (P<0.01) and tibiae (P<0.001). The polar moment of inertia (an estimate of the ability of the structure to resist torsional loading) was decreased in all samples but only significant in 6-week-old tibiae (P<0.05). Similar results were observed in female mice (data not shown). The percentage closed porosity (a measure of the connectivity of the pores) in the cortical bone was also reduced in *Enpp1^−/−^* mice at 22 weeks of age in both femurs (P<0.05) and tibiae (P<0.001). Interestingly, bone mineral density was significantly increased in the femurs and tibia of 22-week-old *Enpp1^−/−^* mice (P<0.05). However, given the reduction in cortical thickness and area, it is possible that there is an overall reduction in the mineral content of the cortex of the *Enpp1^−/−^* mouse.

**Table 3:** MicroCT analysis of cortical bone in male wild-type and *Enpp1^−/−^* mice.

Bone	Age(weeks)	Genotype	% BV/TV	BMD (g/cm^3^)	Cortical Thickness (µm)	Cortical Area(mm^2^)	Closed Porosity (%)	Polar Moment of Inertia (mm^4^)
Tibia	6	WT	88.19 (1.98)	1.15 (0.02)	18.46 (0.36)	0.825 (0.016)	0.087 (0.025)	0.717 (0.037)
		*Enpp1^−/−^*	90.52 (0.39)	1.18 (0.004)	18.44 (0.54)	0.707 (0.055)	0.094 (0.030)	0.512 (0.072)*
	22	WT	98.6 (0.16)	1.31 (0.01)	32.00 (0.34)	1.01 (0.034)	0.113 (0.012)	0.777 (0.06)
		*Enpp1^−/−^*	98.36 (0.10)	1.33 (0.01)*	28.99 (0.43)***	0.778 (0.028)***	0.035 (0.005)***	0.615 (0.043)
Femur	6	WT	93.19 (0.62)	1.21 (0.01)	23.21 (0.41)	0.986 (0.048)	0.091 (0.025)	0.333 (0.037)
		*Enpp1^−/−^*	92.16 (0.44)	1.20 (0.01)	21.41 (0.59)*	0.881 (0.13)	0.069 (0.012)	0.249 (0.032)
	22	WT	98.99 (0.09)	1.38 (0.01)	39.54 (1.05)	0.986 (0.025)	0.058 (0.007)	0.297 (0.046)
		*Enpp1^−/−^*	99.11 (0.14)	1.41 (0.01)**	37.08 (0.62)*	0.865 (0.028)**	0.034 (0.004)*	0.284 (0.354)

6 week femur (WT *n = 7*, *Enpp1^−/−^ n = 6*) and tibia (WT *n = 6*, *Enpp1^−/−^ n = 7*) and 22 week femur (WT and *Enpp1^−/−^ n = 9*) and tibia (WT *n = 8*, *Enpp1^−/−^ n = 9*) were tested. SEM is shown in brackets, significance is denoted by

*P<0.05,

**P<0.01.

***P<0.001.

### The long bones of Enpp1^−/−^ mice have reduced strength and stiffness

**Figure 3:**
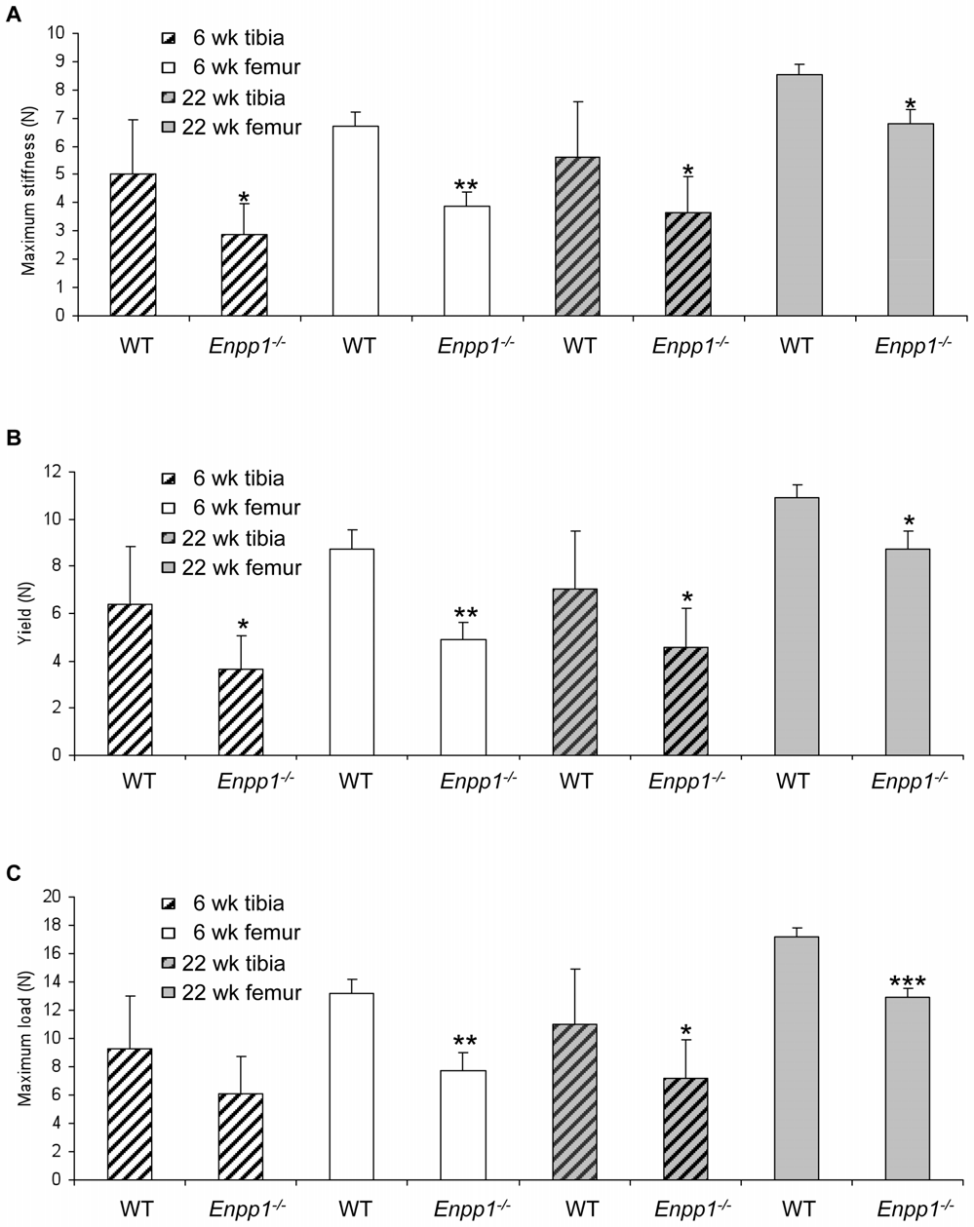
3-point bending shows a reduction in mechanical strength of long bones. Values are shown for tibias and femurs taken from wild-type and *Enpp1^−/−^* mice at 6 weeks and 22 weeks of age. (A) Maximum stiffness was calculated from the point of maximum gradient of a polynomial curve fitted to the load-extension curve. (B) Yield is the point at which the gradient is reduced to 90% of the maximum stiffness. (C) Maximum load was defined as the highest point on the Load-extension curve. Error bars show SEM, significance is denoted by * P<0.05, ** P<0.01 *** P<0.005.

Changes in bone architectural organization in *Enpp1^−/−^* mice are likely to alter the biomechanical properties of the long bones. In order to test this we carried out 3-point bending analysis to determine maximum stiffness (Fig. 3A), yield (Fig. 3B) and maximum load (Fig. 3C) of tibia and femur of 6 and 22-week-old male *Enpp1^−/−^* mice and compared them to wild-type control mice. At 6 weeks of age *Enpp1^−/−^* tibiae showed a significant reduction in maximum stiffness (P<0.05) and yield (P<0.05) and although there was a reduction in maximum load this did not reach significance. The changes in the 6-week-old femora were more marked with significant decreases in maximum stiffness, yield and maximum load (P<0.01). There were similar decreases observed in mice at 22 weeks of age; analysis of the tibiae showed a significant reduction in all parameters tested (P<0.05), whilst the femora showed a significant reduction in maximum stiffness and yield (P<0.05), but a more substantial change in maximum load (P<0.001). The reduced resistance to bending observed in the *Enpp1^−/−^* tibiae and femora is consistent with the reduced cortical bone area and thickness revealed by the µCT analysis. 6-week-old male femurs showed a significant correlation between both cortical thickness and cortical area versus all 3-point bending parameters (P<0.05). A significant correlation between cortical area and maximum load (P<0.05) was observed in 22-week-old femurs. Similarly 6-week-old tibiae showed significant correlations between cortical thickness and both yield and maximum stiffness (P<0.05). However, 22-week-old tibiae showed no correlation between µCT parameters and 3-point bending data. Furthermore, no significant correlations were noted between mechanical parameters and polar moment of inertia.

### Plasma biochemical markers reflect the reduced bone mass observed in Enpp1^−/−^ mice

**Figure 4:**
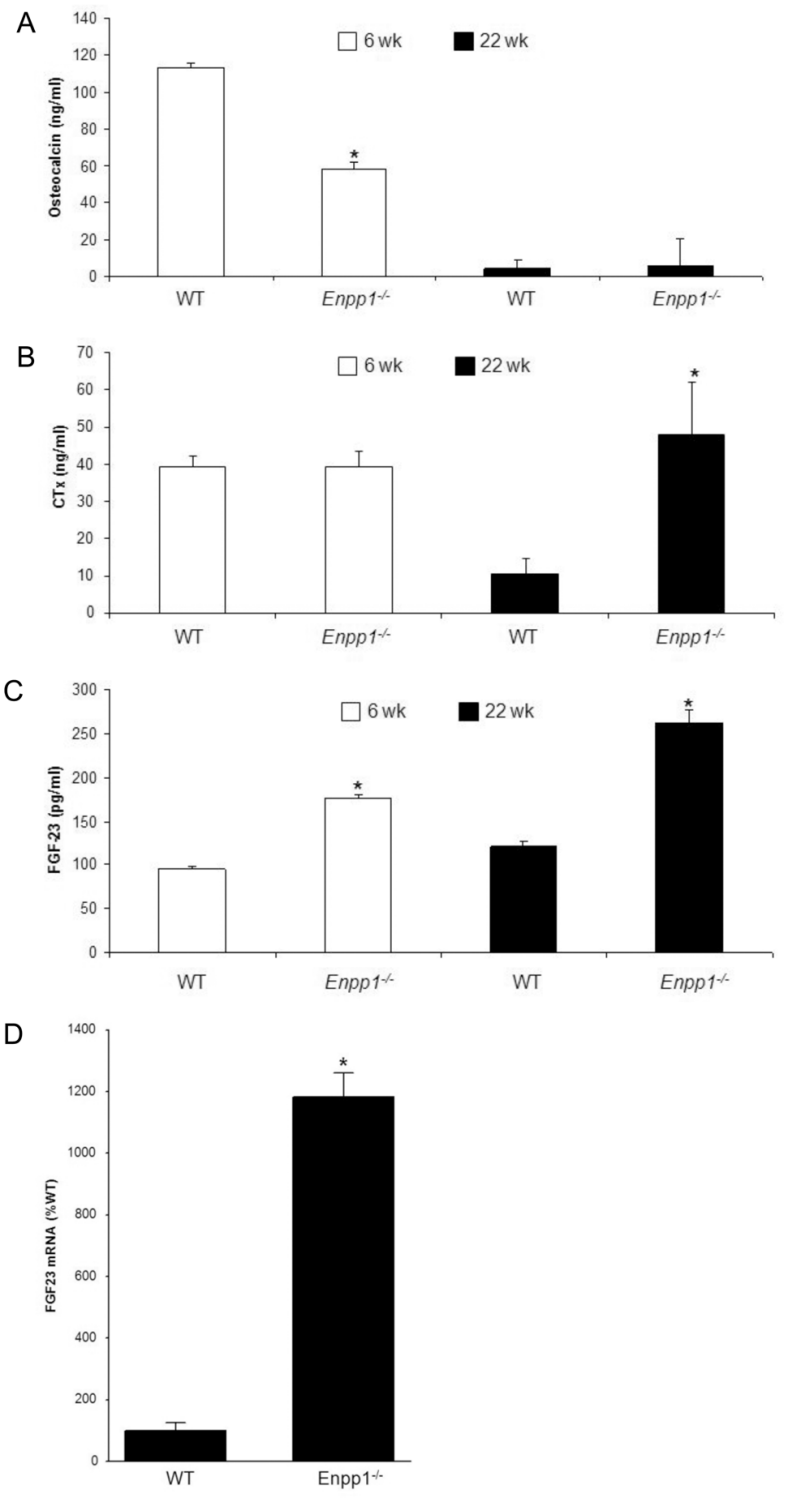
Levels of serum markers and mRNA. (A) Osteocalcin, a marker of bone formation, (B) CTx (RatLaps TM), a marker of bone resorption, and (C) FGF-23 were measured by ELISA in serum samples taken from wild-type and *Enpp1^−/−^* mice at 6 weeks and 22 weeks of age. (D) *Fgf-23* mRNA expression was assessed by RT-qPCR in calvarial bone of wild-type and *Enpp1^−/−^* mice at 12 weeks of age. Error bars show SEM, significance is denoted by *P<0.05, ** P<0.01 *** P<0.005.

The level of osteoblast and osteoclast activity was assessed by ELISA analysis on serum taken from 6 and 22-week-old male *Enpp1* ^−/−^ and wild-type mice. Reduced plasma concentration of osteocalcin, a marker of bone formation, was observed in the male *Enpp1* ^−/−^ mice at 6 weeks of age (96%; P<0.05) (Fig. 4A). By 22 weeks of age, plasma concentrations of osteocalcin had dropped to baseline levels in all mice. It was therefore difficult to observe any potential differences between genotypes in the already low osteocalcin levels observed in the mature animals. Plasma concentrations of CTx, a marker of bone resorption, were unchanged at 6 weeks of age. Interestingly, a reduction in CTx levels, possibly due to a reduction in resorptive activity associated with the cessation of growth, was observed in 22-week-old WT mice. However, the 22-week-old *Enpp1^−/−^* mice did not show this age dependent reduction in CTx levels, and therefore CTx levels in these mice were significantly higher than their WT counterparts (354%; P<0.05) (Fig. 4B). These results suggest that the reduced bone mass observed in the trabecular compartments of the tibia and femur are associated with reduced bone formation in juvenile *Enpp1* ^−/−^ mice and a maintained level of bone resorption in the adult *Enpp1* ^−/−^ mice.

Changes in circulating FGF-23 have been observed in humans with mutant *Enpp1* gene. To determine whether the lack of NPP1 activity had an effect on circulating FGF-23 in mice we carried out an ELISA analysis on serum samples taken from 6 and 22-week-old male *Enpp1* ^−/−^ and wild-type mice. These data showed that there was a significant increase in circulating FGF-23 at both 6 and 22 weeks of age (P<0.05; Fig. 4C), confirming that there is a link between FGF-23 serum levels and NPP1 expression. *Fgf-23* expression is increased in bone of other mutations of genes affecting mineralization, including *Phex*, *Dmp1* and *Ank*. To determine if the increased circulating FGF-23 levels were due to increased *Fgf-23* gene transcription in *Enpp1* ^−/−^ mice, we quantified *Fgf-23* mRNA expression by RT-qPCR. We found that *Fgf-23* mRNA expression in calvarial osteoblasts was 12-fold greater in *Enpp1^−/−^* mice compared to wild-type littermates (Fig. 4D).

A detailed analysis of plasma biochemistry was undertaken on samples taken from female *Enpp1* ^−/−^ mice at 22 weeks of age. Significant reductions in circulating levels of calcium (7%; P<0.05) and P_i_ (16%; P<0.01) were observed in *Enpp1* ^−/−^ mice, which may be a consequence of increased FGF-23 expression dysregulating calcium/phosphate homeostasis. Increased TNAP activity (116%; P<0.01) was observed in *Enpp1* ^−/−^ mice, suggesting a compensatory mechanism to increase bone formation in the absence of NPP1. No effect of genotype on levels of total protein, albumin, globulin, potassium, sodium, bile acids, cholesterol, creatinine, alanine aminotransferase or non-esterified fatty acids were recorded (Table 4). A significant increase in plasma creatine kinase activity (239%; P<0.05; Table 4), a known marker of muscle degradation, was noted.

**Table 4:** Measurements taken of blood serum biochemistry of female 22 week old wild-type and *Enpp1^−/−^* mice.

Analyte	*WT*	*Enpp1^−/−^*
Calcium (mmol/L)	2.31 (0.05)	2.15 (0.05)*
Inorganic phosphate (mmol/L)	2.42 (0.06)	2.03 (0.10)**
Alkaline phosphatase (IU/L)	38.11 (5.65)	82.50 (12.28)**
Creatine kinase (IU/L)	271.22 (34.30)	919.40 (278.89)*
Sodium (mmol/L)	151.0 (0.75)	150.5 (2.05)
Potassium (mmol/L)	8.00 (0.29)	9.45 (0.60)
Non-esterified fatty acids (mmol/L)	0.92 (0.20)	1.05 (0.07)
Total protein (g/L)	51.82 (1.43)	56.95 (2.09)
Albumin (g/L)	28.72 (1.07)	30.13 (0.85)
Globulin (g/L)	23.10 (0.53)	26.82 (1.84)
Bile acids (µmol/L)	80.28 (36.78)	53.31 (19.32)
Cholesterol (mmol/L)	2.11 (0.30)	2.15 (0.18)
Creatinine (µmol/L)	43.78 (1.41)	41.9 (0.97)
Glucose (mmol/L)	10.39 (0.54)	10.88 (0.57)

SEM is shown in brackets, significance is denoted by

*P<0.05,

**P<0.01.

*** P<0.005.

Given that expression of NPP1 has been observed in macrophages [43], analysis of whole blood samples was undertaken to determine whether *Enpp1* ablation is associated with an altered hematological profile. No significant differences were recorded in the number of platelets, lymphocytes, monocytes, neutrophils or eosinophils in the peripheral blood of *Enpp1^−/−^* mice (data not shown). Therefore these data indicate that the severe phenotypic abnormalities observed in the *Enpp1^−/−^* mice do not impact on the hematological profile.

### Histomorphometric analysis shows no change in osteoclast number

Histomorphometric analysis carried out on the tibiae of 22-week-old mice corroborated the µCT data showing a significant reduction in %BV/TV (*Enpp1^−/−^*: 2.2+/−0.59; wild-type: 5.96+/−1.02; P<0.05, n = 4). No significant difference in osteoclast surface/bone surface (*Enpp1^−/−^*: 31.42%+/−5.3; wild-type: 20.69%+/−2.7) or osteoclast number (*Enpp1^−/−^*: 46.63+/−8.6; wild-type: 44.4+/−6.8) was observed in *Enpp1^−/−^* mice. This suggests that the increase in resorption measured by the CTx assay (Fig. 4B) may be due to a greater level of osteoclast activity rather than an increase in osteoclast numbers. Interestingly, a significant increase in osteoblast surface/bone surface (Ob.S/BS) was seen when 22-week-old *Enpp1^−/−^* (39.6+/−7.8) and wild-type (14.7+/−1.8) mice were compared (n = 4; p<0.05). When considered together with the osteocalcin ELISA data (Fig. 4A) these data suggest that there is impaired osteoblast function in the *Enpp1^−/−^* mice.

## Discussion

Impaired HA deposition results in bone frailties such as osteomalacia, rickets and hypophosphatasia. The latter is an inborn-error-of-metabolism, which results from hypomorphic mutations in the TNAP gene, and provides the best evidence of the importance of TNAP for bone mineralization [44]. This lack of TNAP activity results in an excess of its substrate, PP_i_, which is a recognised inhibitor of the mineralization process [19]. Conversely a deficiency in PP_i_ results in ectopic calcification and soft tissue mineralization [7], [28]. Mice lacking NPP1 have severe mineralization defects, which are associated with abnormally low PP_i_ levels [7], [27], [28]. These mice (*Enpp1^−/−^*) are, therefore, a valuable tool with which to understand more fully the role of NPP1 in controlling physiological and pathological mineralization. This present study represents the first detailed evaluation in the adult mouse of the dramatic effects of *Enpp1* ablation on soft tissue calcification and hyperstosis of vertebrae and joints. These data confirm and extend previous reports [9], [45]–[47], and support the role of NPP1 as a critical regulator of mineralization through the production of PP_i_ [28], [47].

Our data indicate that *Enpp1^−/−^* mice have reduced trabecular bone mass and cortical thickness of both the tibia and femur. These changes in bone architecture are consistent with altered markers of bone formation and resorption and explain reduced mechanical properties. This is likely to be a direct effect of lack of NPP1 activity, but the noted reduction in body weight will reduce the loading on the bones and thus may have an effect on their structure. Of particular interest was our observation that by 22 weeks of age the male *Enpp1^−/−^* mice had shorter femurs but longer tibiae compared to wild-type controls. This opens the possibility that changes in structure due to different effects of loading may be occurring, and requires further investigation.

Interestingly, a small but significant increase in cortical BMD was observed at 22 weeks of age in the femur and tibia of *Enpp1* ^−/−^ mice. This increased BMD appears to be a result of a reduction in cortical thickness. This trait appears to be age dependent as BMD was normal in 10 day old *Enpp1^−/−^* mice [28] and similarly BMD and cortical thickness were unaltered in the long bones of 6 week-old *Enpp1^−/−^* mice of this present study. It may also be worth considering that as the *Enpp1^−/−^* mice show mineralization of the vasculature, thus the increase of cortical BMD and percentage closed porosity may be influenced by mineralization of the vessels within the pores of the cortical bone. Further studies are required to address this.

Previous evaluation of the mineralization of bones from 10-day-old *Enpp1^−/−^* and [*Enpp1^−/−^*; *Akp2^−/−^*] double knockout mice indicated that the effects of *Enpp1* ablation on an *Akp2* null background is site-specific [28]. Thus, in contrast to the normalization of the degree of mineralization seen in the joints, calvaria, vertebrae and soft tissues as a consequence of ablating both NPP1 and TNAP function, the long bones of these double knockout mice appeared to remain hypomineralized. This study suggested that hypomineralization observed in the tibia and femur of *Enpp1* ^−/−^ mice may be related to relatively low levels of endogenous NPP1 expression throughout the long bones when compared to the calvaria [28]. Thus, in long bones, the complete deletion of NPP1 activity would further reduce extracellular PP_i_ to abnormally low levels. This would result in insufficient PP_i_ substrate for TNAP to generate P_i_ for normal mineral formation. Interestingly, it has recently been shown that NPP1 can regulate osteoblastic gene expression, and control cellular differentiation, in calvarial osteoblasts independent of PP_i_ and P_i_ [48]. Histomorphometric analysis indicated that there was a significant increase in osteoblast surface/bone surface in tibiae of *Enpp1* ^−/−^ mice. In spite of the increase in osteoblast surface, osteocalcin levels were similar to those in WT mice, suggesting that disruption of *Enpp1* decreases the activity of individual osteoblasts. Furthermore, an accumulation of nucleotide triphosphates due to lack of hydrolysis by NPP1 [49] may have a downstream affect on bone remodelling through purinergic signalling [50].

We also provide further evidence for the severe hypermineralization of the soft tissues including significant arterial calcification in adult *Enpp1* ^−/−^ mice. Notably, significant deposition of calcium in the cortex of the kidney was observed for the first time. These studies also showed that *Enpp1^−/−^* mice had severe hyperostosis of the vertebrae and disorganisation and excessive bone production in the femorotibial joint. These data are consistent with previous reports of an association of an osteopenic phenotype with *Enpp1* ablation, where hypermineralization of the soft tissue, and certain skeletal sites, was observed [28]. This study went on to show that calcified nodule formation and mineral deposition are inhibited to a higher extent in osteoblasts isolated from *Enpp1^−/−^* bone marrow than calvarial osteoblasts isolated from the same animal. This indicates that loss of NPP1 activity affects skeletal sites in a site-specific manner [28].

As NPP1 is known to be important mediator of insulin signalling in various tissue types including adipose and muscle [51], the effects of glucose regulation may also contribute to the hypomineralization observed in the long bones. Our data show that *Enpp1* null mice display significantly reduced body weight in juvenile and adult mice. Long bone lengths and growth plate widths were unchanged at 6 weeks of age but a significant reduction in body weight was observed in these mice. This may indicate reduced fat accumulation associated with increased insulin sensitivity in mice lacking NPP1, given the previously reported observations of insulin resistance and glucose intolerance in mice with over-expression of hepatic NPP1 [52]. Furthermore, administration of calcitonin to the *ttw/ttw* mouse ameliorates the osteopenic effect. Calcitonin's main biological function is to inhibit osteoclast activity [53]. This suggests that in *ttw/ttw* mice - and possibly *Enpp1* ^−/−^ mice, given the observed increase in circulating CTx - a state of increased osteoclastic activity leads to bone loss at certain sites. This increased osteoclastic activity could in turn lead to increased osteocalcin decarboxylation, favouring pancreatic beta cell proliferation, insulin secretion, insulin sensitivity and energy expenditure [54], [55]. The regulation of insulin signalling in bone by NPP1 requires further investigation.

Our data show that *Enpp1^−/−^* mice maintain similar levels of osteoclast activity at 6 and 22 weeks of age, whereas WT mice show reduced bone resorption with advancing age which is consistent with the attainment of the adult skeleton. Surprisingly we do not see a concomitant rise in osteoclast number or osteoclast surface/bone surface, indicating an upregulation of osteoclast function. We have also described an increase in circulating levels of creatine kinase, a cytoplasmic enzyme released during tissue turnover. This is most likely to be due to skeletal muscle disruption in the areas of hyperostosis. However, it has been reported that the presence of brain-type creatine kinase (Ckb) is greatly increased during osteoclastogenesis and that reduction in Ckb expression using RNAi technology resulted in reduced bone loss in both rat and mouse models [56]. Furthermore, the presence of creatine kinase in matrix vesicles isolated from femurs of chicken embryos suggests it has an active role in bone mineralization [57]. These studies suggest that osteoclast function in the *Enpp1* ^−/−^ mice may be affected by an increase in creatine kinase levels.

Elevated FGF-23 circulating levels and expression in bone adds to a growing number of single gene mutations whose activation impairs bone mineralization and leads to increments in *Fgf-23* gene transcription [58]. FGF-23 is known as a phosphaturic hormone that controls phosphate homeostasis, calcium homeostasis and bone mineralization. FGF-23 binds to FGF receptors (mainly FGFR1) and the co-receptor KLOTHO in the kidney and promotes excretion of Pi, which leads to reduced serum Pi [59], [60] and stimulation of Cyp24 and inhibition of Cyp27b1 in the kidney to reduce circulating 1,25(OH)2D levels. Therefore the decreases in circulating calcium and phosphate levels in *Enpp1* ^−/−^ mice are consistent with excess FGF-23. Regardless, our findings in *Enpp1^−/−^* mice are consistent with human genetic studies that have recently shown that *Enpp1*, if mutated, causes hypophosphatemic rickets resulting from increased FGF-23 levels [17]. The mechanism whereby *Fgf-23* gene transcription in bone is stimulated by *Enpp1* inactivation is not defined by our studies, however, recent data indicate alterations in matrix mineralization caused by other single gene mutations in osteoblasts leads to stimulation of *Fgf-23* expression via FGF receptor activation [61]. Further studies will be needed to determine if the increase in FGF-23 observed in *Enpp1^−/−^* bone is intrinsic and due to pathways similar to *Phex* and *Dmp1* mutations [11], [15] or as a result of distinct signalling pathways. Observed increases in serum FGF-23 levels may regulate the *Enpp1* ^−/−^ bone phenotype through the bone-kidney axis or through local effects on bone cells. There is also controversial evidence that FGF-23 may directly affect skeletal mineralization, independent of phosphate homeostasis [62], which further confounds the interpretation of the bone phenotype in *Enpp1^−/−^* mice. Alternatively, reductions in PP_i_ concentrations, the precursor to P_i_, could result in local reductions in P_i_ concentrations in the extracellular matrix required for normal mineralization. More detailed studies examining the roles of increased FGF-23 levels on bone homeostasis in *Enpp1* ^−/−^ mice through local or systemic effects are needed.

In summary, our data demonstrate that *Enpp1^−/−^* mice are characterized by severe disruption to the structural and mechanical properties of long bones, the severity of which increases with age. Furthermore, dysregulation of calcium/phosphate homeostasis and hypercalcification in joints and soft tissues confirms that NPP1 plays important roles in calcium and phosphate regulation and repression of soft tissue mineralization, as well as maintaining skeletal structure and function.
