# Correction: Altered Bone Development and an Increase in FGF-23 Expression in *Enpp1^−/−^* Mice

**DOI:** 10.1371/annotation/8f71d7e8-d81d-4878-bf14-79a313a7810b

**Published:** 2012-06-13

**Authors:** Neil Charles Wallace Mackenzie, Dongxing Zhu, Elspeth M. Milne, Rob van 't Hof, Aline Martin, Darryl Leigh Quarles, José Luis Millán, Colin Farquharson, Vicky Elisabeth MacRae

There was an error in the sixth author's name. Leigh Darryl Quarles is correct. 

